# Matrix metalloproteinases as biomarkers and therapeutic targets in colitis-associated cancer

**DOI:** 10.3389/fonc.2023.1325095

**Published:** 2024-01-15

**Authors:** Natalia Sampaio Moura, Alyssa Schledwitz, Madeline Alizadeh, Seema A. Patil, Jean-Pierre Raufman

**Affiliations:** ^1^Department of Medicine, Division of Gastroenterology and Hepatology, University of Maryland School of Medicine, Baltimore, MD, United States; ^2^The Institute for Genome Sciences, University of Maryland School of Medicine, Baltimore, MD, United States; ^3^Medical Service, Veterans Affairs Maryland Healthcare System, Baltimore, MD, United States; ^4^Marlene and Stewart Greenebaum Cancer Center, University of Maryland Medical Center, Baltimore, MD, United States; ^5^Department of Biochemistry and Molecular Biology, University of Maryland School of Medicine, Baltimore, MD, United States

**Keywords:** matrix metalloproteinases, colitis-associated cancer, colorectal cancer, inflammatory bowel disease, tissue inhibitors of metalloproteinases, enzymes, cell invasion, biomarkers

## Abstract

Colorectal cancer (CRC) remains a major cause of morbidity and mortality. Therapeutic approaches for advanced CRC are limited and rarely provide long-term benefit. Enzymes comprising the 24-member matrix metalloproteinase (MMP) family of zinc- and calcium-dependent endopeptidases are key players in extracellular matrix degradation, a requirement for colon tumor expansion, invasion, and metastasis; hence, MMPs are an important research focus. Compared to sporadic CRC, less is known regarding the molecular mechanisms and the role of MMPs in the development and progression of colitis-associated cancer (CAC) − CRC on a background of chronic inflammatory bowel disease (IBD) − primarily ulcerative colitis and Crohn’s disease. Hence, the potential of MMPs as biomarkers and therapeutic targets for CAC is uncertain. Our goal was to review data regarding the role of MMPs in the development and progression of CAC. We sought to identify promising prognostic and therapeutic opportunities and novel lines of investigation. A key observation is that since MMPs may be more active in early phases of CAC, using MMPs as biomarkers of advancing neoplasia and as potential therapeutic targets for adjuvant therapy in those with advanced stage primary CAC rather than overt metastases may yield more favorable outcomes.

## 1 Introduction

Enzymes comprising the 24-member matrix metalloproteinase (MMP) family of zinc- and calcium-dependent endopeptidases play key roles in cancer progression by degrading the extracellular matrix, an obstacle to tumor expansion, invasion, and metastasis. Notably, differential induction of specific MMPs has been reported depending on the cancer type examined and how the neoplastic cells are stimulated. For example, more than a decade ago, our research team reported that stimulating human colon cancer cells with muscarinic receptor agonists resulted in robust, selective induction of MMP-1, -7, and -10 (1). Subsequent work indicated that blocking muscarinic receptor activation or the activity of MMP-1, whose expression correlates with advanced colon cancer stage, tumor metastasis, and reduced survival (2–4), abolished acetylcholine-induced colon cancer cell invasion (5). Moreover, we showed that muscarinic receptor agonist-induced MMP-1 expression is mediated by potentiating crosstalk between different post-muscarinic receptor signaling cascades (6).

Despite the great interest in understanding the role MMPs play in the progression of sporadic colorectal cancer (CRC), relatively little attention has been paid to their role in colitis-associated colon cancer (CAC). This area of investigation is also relevant to our focus on the role of muscarinic receptors and ligands in CRC (7–9) – our work and that of others has demonstrated that muscarinic receptor activation may play an important role in the progression of inflammatory bowel disease (IBD, primarily Crohn’s disease and ulcerative colitis (UC)) (10, 11). Moreover, current work highlights the unique molecular differences between CAC compared to non-colitis-associated CRC (12), and the strong involvement of MMPs in regulating colitis and intestinal permeability (13, 14). Hence, understanding the precise role that MMPs play in the genesis and progression of CAC, and how these differ from their roles in sporadic CRC, may reveal novel mechanistic insights, but more importantly, identify MMPs as promising biomarkers of CAC progression and therapeutic targets.

For these reasons, in this review, we aim to provide a thorough appraisal of MMP’s functions, their role in the progression from chronic colitis to CAC, and an overview of the application of MMP inhibitors in clinical therapy. We searched the literature for English language publications between 1962 and June 30, 2023, using keywords relevant to this search (IBD, inflammation, matrix metalloproteinases, colitis-associated cancer, extracellular matrix, dysplasia, biomarkers), alone and in combination, in the following public databases: PubMed, ScienceDirect, and BioMed Central. Included publications were reviewed critically, and the results summarized in the following text and tables. We believe the results of this approach yielded important information that can be used to develop preliminary insights, but perhaps more importantly, identify areas worthy of further basic and clinical investigation.

## 2 Matrix metalloproteinases

### 2.1 Subgroups of matrix metalloproteinases

The extracellular matrix (ECM), comprised of fibers constructed from collagen, elastin, laminin, fibronectin, proteoglycans, glycoproteins, and polysaccharides, serves as a meshwork for tissue development and maintenance, and plays a key role in cell migration and adhesion (15). Matrix metalloproteinases (MMPs) represent a 24-member family of zinc and calcium endopeptidases that degrade structural macromolecules within the ECM. Non-ECM substrates for MMPs include proenzymes and proinflammatory cytokines, chemokines, and bacteria (16). MMPs are numbered from 1 to 28; MMP-4, -5, and -6, subsequently recognized to be MMP-1, -2, and -3, respectively, are omitted, and MMP-18 was likewise later recognized to be MMP-19 (17–19). The first MMP was isolated and characterized in 1962 in a tadpole tail (20) and 26 years later, human fibroblast stromelysin and type IV collagenase were discovered and found to possess a wider range of substrate specificities (21, 22). Interest within the scientific community continued to evolve as additional MMPs were discovered and their role in disease, particularly cancer, became apparent. The late 20^th^ century and early 21^st^ century witnessed important advances in the characterization of MMPs, followed by investigation into their roles as potential biomarkers and therapeutic targets. This is especially due to advances in research tools, evolving from protein isolation and purification to more advanced tools, e.g., gene cloning, transgenic mice, immunofluorescence microscopy, and single cell RNA expression profiling.

**Figure 1:**
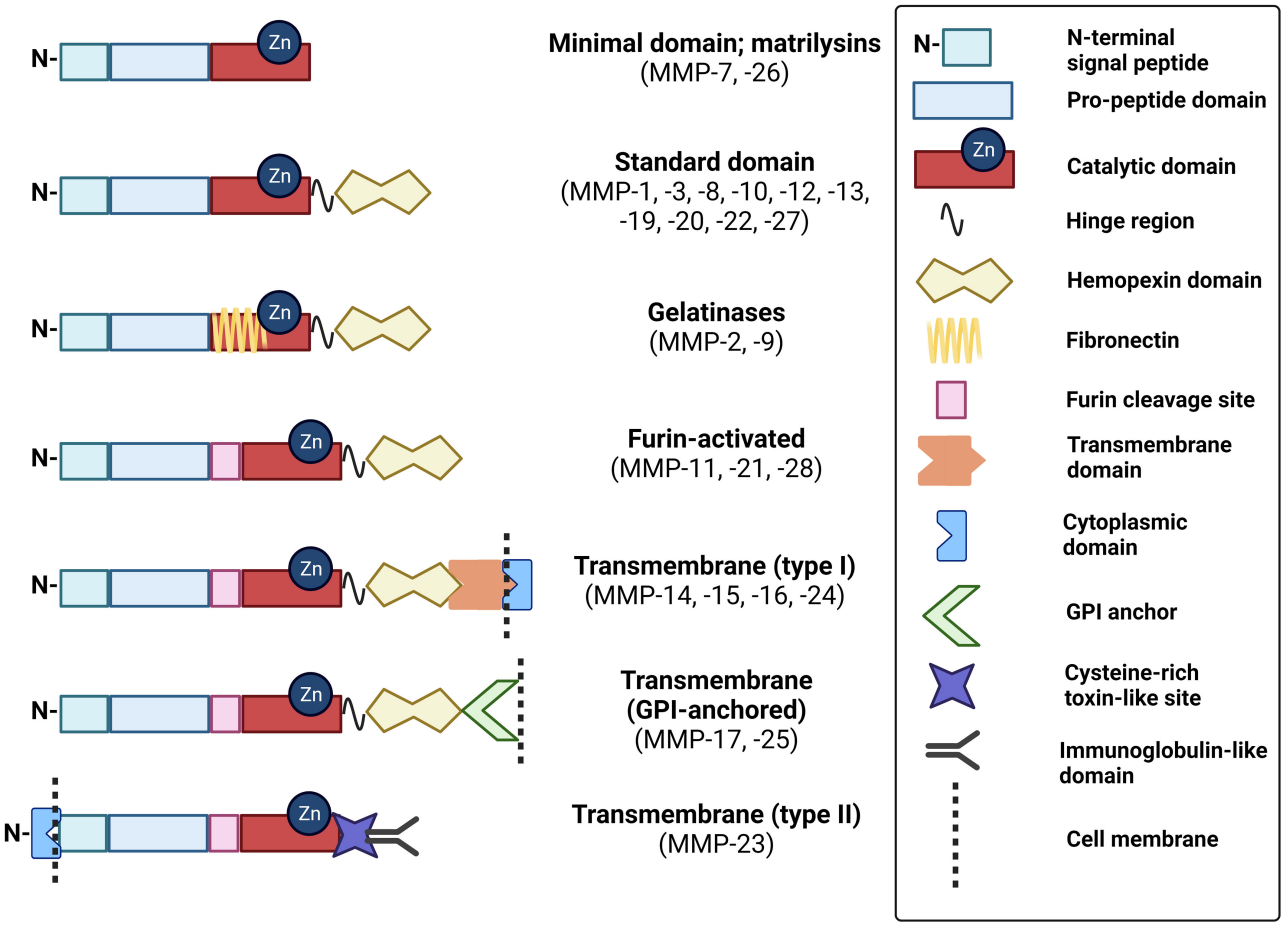
Schematic of MMP domain structures. These schematics of MMP subtypes illustrate the relative distribution of key domains that modulate their structure and function. GPI, glycophosphatidylinositol. Created with BioRender.com.

MMPs are classified as collagenases, gelatinases, stromelysins, matrilysins, metalloelastase, enamelysin, epilysin, and membrane-type MMPs. The major difference between these enzymes is domain organization and substrate specificity, illustrated in **Figure 1**. As anticipated from a family of proteins, MMP structural similarity is high. MMPs share three basic domains: the N-terminus domain, pro-domain near the active site, and catalytic domain. Upon activation, the N-terminus and pro-domain are cleaved. Whereas matrilysins (MMP-7 and -26) have this basic organization, collagenases (MMP-1, -8, -13), stromelysins (MMP-3, -10), metalloelastase (MMP-12), enamelysin (MMP-20), and MMP-22 and -27 have an additional hemopexin-like domain close to the C- terminus, which binds and helps to orient the substrate correctly. MMPs differ broadly in substrate specificity; collagenases cleave peptide bonds present in collagen, stromelysins cleave a wider range of substrates (proteoglycan, gelatin, fibronectin, lamin, and collagen), metalloelastases degrade elastin, and, as its name implies, enamelysin modulates enamel formation. Gelatinases (MMP-2, -9) have fibronectin repeats around the catalytic site and primarily target gelatin and type IV collagen. Membrane-bound MMPs possess either an additional transmembrane peptide domain (MMP-14, -15, -16, -24), or glycophosphatidylinositol (GPI)-anchoring domain (MMP-17, -25) and have a basic amino acid motif at the C-terminus, that is recognized and cleaved for activation by proprotein convertases. Notably, MMP-23 contains a type II transmembrane domain and, instead of the hemopexin domain common to other MMPs, possesses a small toxin-like domain and immunoglobulin-like cell adhesion molecule domain that informs its unique properties in modulating voltage-gated potassium activity and interactions with components of the ECM and signaling molecules (23, 24). An overview of the MMP substrates and physiological functions unrelated to CAC development are included in **Table 1**.

**Table 1:** Overview of MMP substrates and physiological functions with currently unknown roles in the development of CAC.

MMP	Alternate names	Substrates	Role	Refs
MMP-12	Metalloelastase, macrophage elastase	Elastin, type IV collagen, laminin, fibronectin	Macrophage recruitment; adipose tissue expansion; directly bactericidal	(14, 25–27)
MMP-15	MT2-MMP	Collagen, fibronectin, laminin, gelatin, pro-MMP-2 and -13	Cell migration, cytotrophoblast invasion	(28–30)
MMP-16	MT3-MMP	Fibrin, pro-MMP-2	Cell migration	(31)
MMP-17	MT4-MMP	Fibrinogen, pro-TNF, ADAMTS4, α-2-macroglobulin	Cell proliferation, angiogenesis, limb development	(32, 33)
MMP-19	RASI-1, stromelysin-4	Collagen I, IV, gelatin, fibronectin	Wound healing, epithelial proliferation	(34–36)
MMP-20	Enamelysin	Amelogenin	Tooth enamel development	(37)
MMP-21	N/A	Gelatin, a1-antiptrypsin, aggrecan	Embryogenesis, tumor progression	(38–40)
MMP-22	N/A	Gelatin, casein	Upregulated in coronary heart disease	(41, 42)
MMP-23	N/A	Gelatin	Modulates voltage-gated K+ channel trafficking	(24, 43)
MMP-24	MT5-MMP	Fibronectin, pro-MMP-2	Modulates neural stem cell proliferation and promotes neuroinflammation	(44–46)
MMP-25	MT6-MMP	Gelatin, collagen type IV, fibronectin, laminin, pro-MMP-2	Respiratory burst of phagocytes, IL-18 secretion, tumor progression	(33, 47, 48)
MMP-26	Matrilysin-2, endometase	Fibrinogen, fibronectin, vitronectin, denatured collagen	Enterocyte migration, tumor progression	(35, 49)
MMP-27	N/A	Unknown	Regulation of menstruation	(50)
MMP-28	Epilysin	Casein	Role in tissue homeostasis	(35, 51, 52)

TNF, tumor necrosis factor; CAC, colitis-associated cancer; Refs, references; N/A, not applicable.

Due to their involvement in a multitude of biological processes, dysregulated MMP expression or function can broadly impact health and disease. MMP dysregulation is evident in fibrotic diseases, vasculopathies, and tissue degradation that promote cancer progression, ulceration, rheumatoid arthritis, osteoarthritis, periodontal diseases, among a host of other conditions. For example, poor wound healing is characterized by increased MMP-9 expression, with significantly higher levels detected in unhealed diabetic foot ulcers (53). As a consequence of their roles in degrading the pericellular matrix, activating other MMPs, and stimulating epidermal growth factor (EGF) receptor (EGFR)-dependent cell motility and growth, many MMPs, including collagenases, gelatinases, matrilysin, metalloelastase, and membrane-bound MMPs, promote neoplasia – e.g., cellular invasion and metastasis (23). By modulating chemokine and cytokine activity and bacterial clearance, MMPs are involved in initiating acute inflammatory responses after tissue injury. Nonetheless, MMPs can have conflicting functions that either attenuate or augment inflammation. For example, although MMP-7 expressed in non-inflamed epithelium modulates homeostasis, it can also act on syndecan-1 after its release by endothelial cells, to establish a local post-injury chemokine gradient that activates FAS ligand to trigger apoptosis (54, 55).

### 2.2 Cellular regulation of matrix metalloproteinase activity

**Figure 2:**
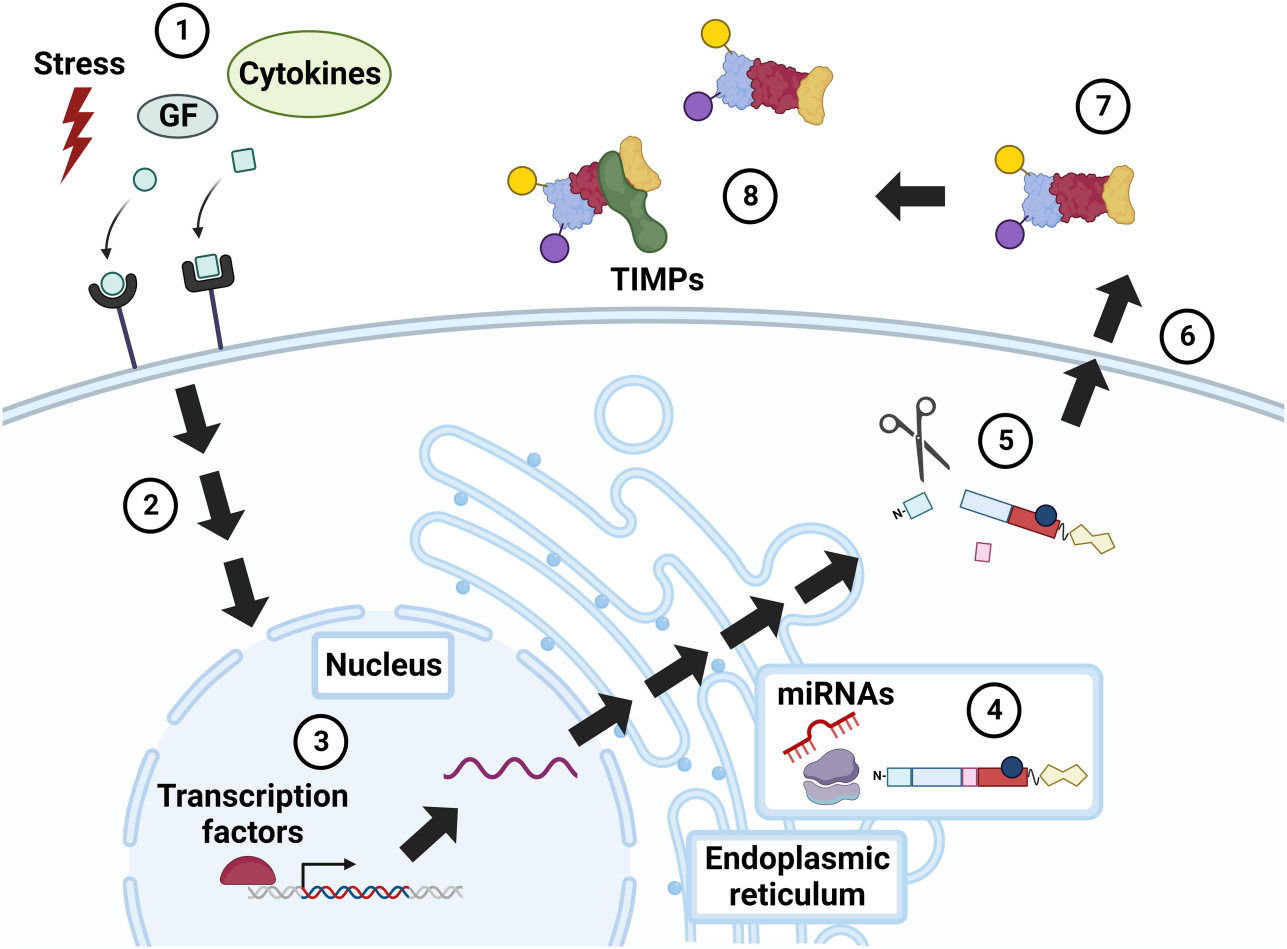
Regulation of MMP activity. Constitutive MMP expression is augmented in response to external stimuli, e.g., physiochemical stress, growth factors (GF), and cytokines (1), that incite intracellular signaling via mitogen activated protein kinase (MAPK), NF-kB, Smad, and others (2). MMP expression is further modulated by transcription factors, notably AP-1 (3). MMP RNA is translated into a pre-propeptide (4), a process that can be up- and down-regulated by microRNAs (miRNAs). Cleavage of the N-terminal signal peptide in the endoplasmic reticulum (ER) yields the pro-MMP zymogen, which may undergo intracellular (e.g., cleavage by furin) and extracellular (e.g., cleavage by plasmin) modification (5). Cellular release of MMPs is also regulated by stress, growth factors, and cytokines (6). Extracellular MMPs are activated by post-translational modifications (7), or by the action of other, membrane-bound MMPs. MMP activity is also modulated by complexes formed with inhibitors, i.e., TIMPs and α2-macroglobulin (8). GF, growth factors; TIMP, tissue inhibitor of metalloproteinase. Created with BioRender.com.

Because they play such key roles in modulating cell functions, MMP activity must be tightly regulated at the transcriptional, post-transcriptional, and proteomic levels (**Figure 2**). MMPs are produced and secreted by many different cell types, including dermal fibroblasts, osteoblasts, and endothelial and inflammatory cells. Typically located peri- or extracellularly, both secreted and membrane-bound MMPs can also localize to intracellular sites (56). MMP secretion can be stimulated by cytokines, including proinflammatory cytokines (e.g., interleukins and interferons), growth factors (e.g., EGF and acetylcholine), and physiochemical agents (e.g., heat) (15, 57). Particular cell types evidence signal-dependent activation and repression of MMP gene transcription by processes involving mitogen-activated protein kinase (MAPK)-, nuclear factor-kB (NF-kB)-, and Smad-dependent pathways. Specific transcription factors such as AP-1, PEA3, Sp1, Tcf/Lef-1, and NF-kB serve as cis-acting elements that can bind proximal to the MMP promoter to induce expression. In 1987, AP-1 was the first inducer implicated in MMP-1 expression (58).

MMPs are regulated post transcriptionally by micro(mi)RNAs, a family of short non-coding RNAs that modify gene expression. Dysregulated miRNA activity can alter cancer progression by sustaining proliferative signaling or enhancing invasion and metastasis (59). For example, Wu et al. showed that the tumor suppressor p53 can induce miR-34a expression and, thereby, downregulate MMP-1 and MMP-9, and attenuate cell migration and invasion (60). Sun et al. demonstrated that increased miR-21 expression in serum from patients with CRC and in colon cancer cell lines correlated with increased MMP-2, -9, and -11 expression (61). Other miRNA-MMP-linked interactions impacting CRC progression have been studied, including miR-139-MMP-2, miR-146a-MMP-16 (62, 63).

After transcription, MMPs are synthesized as pre-proenzymes. Following removal of the N-terminal signal sequence during translation, each MMP exists in three forms: the inactive secreted form or zymogen known as pro-MMP, active MMP, and inhibitor-complexed MMP (64, 65). Pro-MMPs are secreted and activated by proteolytic removal of the pro-domain by other MMPs, particularly membrane-type MMPs, serine proteases, plasmin, or furin. Activation via serine proteases can be regulated by inhibition of plasma proteinase inhibitors, such as α1-proteinase (66). Once activated, MMPs possess catalytic activity until they bind to two major types of endogenous inhibitors, α2-macroglobulin and tissue inhibitor of matrix metalloproteinases (TIMPs). α2-macroglobulin, a protease inhibitor abundant in plasma, binds to MMPs extracellularly, allowing for their removal by receptor-mediated endocytosis (67). In turn, TIMPs, a family of four inhibitors (numbered 1-4), regulate processes mediated by MMPs and ADAMs (A Disintegrin And Metalloproteinase), another family of zinc-dependent peptidases related to MMPs (68). Dysregulation of the MMP : TIMP ratio can upregulate active MMP expression and ECM damage (64). Recent data show that TIMPs also modulate biological processes independent of MMP and ADAM activity, adding additional complexity to their impact on tumor proliferation and/or inhibition (69). TIMP-1 preferentially inhibits MMP-1, -3, -7, and -9, and has stimulatory cellular properties (68, 70). TIMP-2 traditionally inhibits MMP-2 and -9, but, in aggressive cancers, TIMP-2 uniquely binds and activates pro-MMP-2 and MT1-MMP to stimulate MAPK/ERK signaling (68, 71). Recently, Li et al. identified elevated TIMP-2 expression in the serum of patients with CRC resistant to 5-fluorouracil (5-FU), a possible biomarker of 5-FU resistance (72). TIMP-3 preferentially inhibits MMP-2 and MMP-9. In mice, TIMP-3 deficiency leads to maladaptive ECM remodeling, cardiomyocyte hypertrophy, and cardiac dysfunction (73). Though less studied, TIMP-4 appears to inhibit MMP-26 preferentially as well as contribute to the proteolysis of a cell surface fatty acid transporter, underlying its role in intestinal lipid absorption (74, 75).

## 3 Colitis-associated colon cancer

### 3.1 Relationships between IBD and colon cancer

IBD is associated with a two- to three-fold increased risk of CRC, a major cause of IBD mortality and need for colectomy (76). The frequency rate of CAC is 1.78% among IBD patients (2.1% for ulcerative colitis and 1.5% for Crohn’s disease), while the rate of CRC is 1.23% in the general population (77). Notably, in line with the decreasing mortality from CRC in the United States, the rates of CAC incidence and mortality also appear to be declining. The latter is likely impacted by the development of a wide array of novel therapeutic modalities targeting the inflammatory cascade, e.g., biologicals targeting tumor necrosis factor and the immune response.

Key mechanistic differences that distinguish CAC from sporadic CRC raise important questions regarding the need for a distinct approach to prevention, surveillance, and management. The driving force behind CAC is chronic inflammation, which amongst other consequences, promotes DNA oxidative damage that may alter the expression and function of genes identified as tumor promotors and suppressors (12). The risk factors most often associated with CAC reflect the influence of chronic inflammation, including the anatomical extent of disease, the severity of histological injury, and the cumulative inflammatory burden (78).

**Figure 3:**
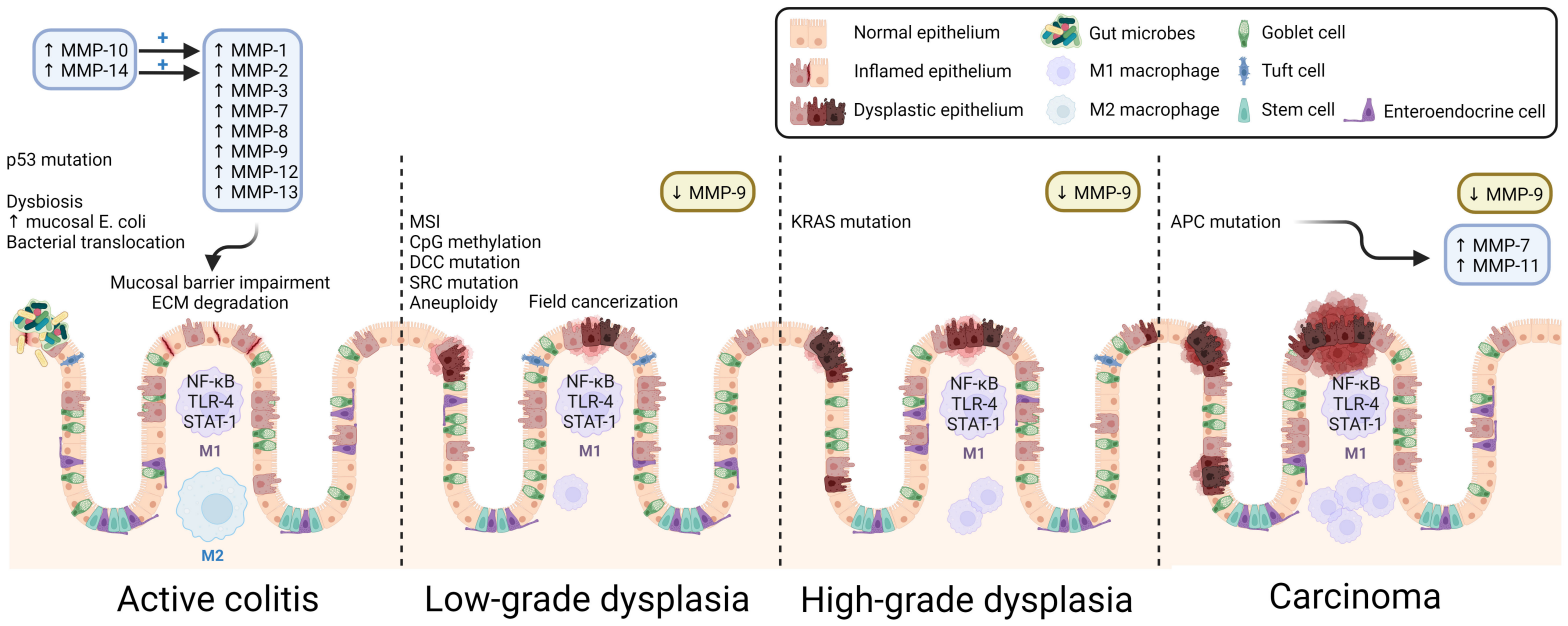
Overview of CAC pathogenesis. Many MMPs are over-expressed, and their activity upregulated in active colitis. MMP-induced mucosal damage, inflammatory dysbiosis, early p53 mutation, and other results of DNA damage promote dysplastic changes. Because the inflammatory insult is widespread, CAC exhibits field cancerization, and multiple malignant foci form in the affected tissue. As dysplasia progresses, the population of colonic macrophages shifts toward the pro-inflammatory M1 macrophage subtype. Despite its membrane-degrading effects in colitis, MMP-9 is consistently found to be downregulated in CAC and acts as a tumor suppressor. ECM, extracellular matrix; MSI, microsatellite instability. Created with BioRender.com.

While the same molecular pathways that contribute to the development of CRC are involved in CAC, including chromosomal and microsatellite instability, the sequence of common gene alterations differs considerably. In contrast to the canonical CRC adenoma-carcinoma sequence, in CAC, p53 mutation is an early event, occurring even before the development of dysplasia (79) (**Figure 3**). APC mutation, an early event in CRC, occurs later in the development of CAC (79). The presence of similar burden of mutations in non-dysplastic mucosa adjacent to CAC suggests a primed field effect, which may explain high rates of synchronous and metachronous dysplasia in the same region (80, 81). These differences may contribute to the difficulty detecting epithelial dysplasia using colonoscopy in those with IBD. While most dysplastic colonic lesions in IBD are visible on high-definition white light endoscopic evaluation, their flatter morphology can have a more subtle appearance than classical adenomatous polyps that precede the development of CRC. Using tools like dye-based and virtual chromoendoscopy can augment the ability to detect dysplasia in CAC (82). Additionally, in IBD, the rapid and recurrent development of dysplasia in primed precancerous fields requires repeated surveillance colonoscopies at shorter intervals than those recommended for screening for sporadic CRC (83). The implementation of such dysplasia surveillance protocols in IBD is also thought to be a key contributor to the decreasing rates of CAC.

In IBD, concomitant primary sclerosing cholangitis (PSC) greatly augments CAC risk. Compared to those with IBD alone, the additional diagnosis of PSC confers an additional three- to five-fold increased risk of developing CAC. The mechanisms leading to this increased risk have not yet been clarified but may differ from those underlying CAC in IBD alone. Proposed factors include altered bile acid metabolism, intestinal or biliary dysbiosis, and systemic immunological dysfunction (84). It is both interesting and puzzling that colonic inflammation itself appears to be less influential, as those with concomitant IBD and PSC who develop CAC often have quiescent clinical, endoscopic, and histologic disease.

Given the many mechanistic differences between CAC and sporadic CRC, as well as the relative paucity of research on the treatment of CAC compared to that of sporadic CRC, CAC presents many challenges to treatment. Management of CAC includes surgical intervention with administration of adjuvant chemotherapy, like treatment of CRC (85, 86). Unfortunately, patients with metastatic CAC fare more poorly than age- and tumor type-matched patients with metastatic sporadic CRC, even when treated with first-line chemotherapy regimens such as FOLFOX or FOLFIRI (87). This underscores the need for improved early disease detection, and for the development of therapies that target not only the carcinogenic milieu of CRC but also CAC’s hallmark inflammatory dysregulation (85). Even the type of underlying IBD may influence disease outcome in CAC, adding additional facets to consider in the search for meaningful CAC biomarkers. For example, individuals with Crohn’s disease had more advanced CAC stage at diagnosis than those with UC, leading to poorer survival outcomes for Crohn’s-associated colorectal cancer (88).

### 3.2 Roles of matrix metalloproteinases in CAC pathogenesis

MMPs are involved in myriad cellular and extracellular processes, including epithelial proliferation, ECM homeostasis, angiogenesis, and response to inflammation. As such, they are a topic of interest in cancer research, particularly CRC research. There is a growing body of literature regarding the roles of MMPs in the relatively more enigmatic CAC, and in pre-cancerous IBD (**Table 2**). Given clear differences in the pathogenesis and clinical features of CAC, compared to non-inflammatory CRC, in the following paragraphs, we summarize the current literature on MMPs in CAC, an underdeveloped focus of investigation.

**Table 2:** Overview of MMPs with known roles in the development of colitis and/or CAC.

MMP	Alternate names	Substrates	Role	Involvement in CAC	Refs
MMP-1	Collagenase-1	Types I-III collagen	Cell migration, reepithelization, TNF activation	Upregulated in UC and CD; involvement in CAC unknown	(89)
MMP-2	Type IV collagenase, gelatinase A	Collagen, gelatin	Neurite outgrowth, cell migration	Assists in epithelial barrier maintenance to prevent initiation of colitis	(22, 89)
MMP-3	Proteoglycanase, stromelysin-1	Proteoglycan, casein, fibronectin, laminin	Adipocyte proliferation, antithrombotic activity, wound healing	Upregulated in UC	(90–92)
MMP-7	Matrilysin-1	Gelatin, laminin, fibronectin, vitronectin, and elastin	CRC metastasis, adipocyte differentiation	Upregulated in IBD-associated dysplasia	(89, 93–96)
MMP-8	Collagenase-2	Fibrils of types I- III collagen	Involved in neutrophilic infiltration	Upregulated in UC	(97, 98)
MMP-9	Gelatinase B	Gelatin, collagen, elastin	Trophoblastic invasion, neo-vascularization, macrophage and neutrophil activation	Mediates inflammation in colitis; attenuates tumorigenesis in CAC	(99–102)
MMP-10	Stromelysin-2	Proteoglycan, casein, fibronectin, laminin	Wound healing	Resolution of inflammation; MMP-10 deletion increases murine CAC burden	(103, 104)
MMP-11	Stromelysin-3	Serpins, e.g., a2-macroglobulin, a1-proteinase inhibitors	Adipocyte de-differentiation, desmoplastic reaction in cancer-adjacent tissue, early tumor invasion	Attenuated CRC cell apoptosis in MMP-11-deficient mice; expression reduced by infliximab treatment in a murine IBD model, suggesting a contributory role in CAC	(105–108)
MMP-13	Collagenase-3	Fibrils of type I-III collagen	Osteoclast and TNF activation	Associated with increased intestinal permeability upon inflammation, and upregulated in colitis	(89, 109)
MMP-14	MT1-MMP	Collagen type I-III, gelatin, CD44, pro-MMP-2, -8, and -13	Cell migration, cytotrophoblast invasion	Upregulated in cancer-associated fibroblasts found in CAC	(65, 96)

TNF, tumor necrosis factor; UC, ulcerative colitis; CD, Crohn**’**s disease; CAC, colitis-associated cancer; IBD, inflammatory bowel disease; Refs, references.

Unlike sporadic colon cancer, CAC arises from chronic inflammation-related DNA damage, with distinct mechanisms and a significant role for immune dysregulation (12, 110). It is not surprising that increasing IBD duration and severity pose progressively increased CAC risk (111). During acute intestinal inflammation, several MMPs are upregulated, most prominently MMP-1, -8, -9, -10, -12, and -13; persistent overactivity of MMPs impairs ECM integrity, which itself can exacerbate IBD, heralding chronic inflammation and pre-cancerous DNA damage (**Figure 1**) (112). Biopsies of IBD-related intestinal epithelial damage (ulcers) reveal increased gene and protein expression of MMP-1, -2, -3, -7, and -9, as well as MMP-10, and -14 that activate MMP-1 and -2 (113, 114). *In vitro* studies revealed that MMP-9 derived from intestinal epithelial cells (but not from neutrophils) plays a key role in the pathogenesis of colitis by inhibiting epithelial cell-ECM interaction and wound healing; MMP-9 knockout mice are more resistant to various types of induced colitis (115, 116).

The roles of MMP-9 in IBD and in CAC are of particular interest. Although MMP-9 appears to facilitate pro-inflammatory signaling in non-cancerous or pre-cancerous IBD, its actions may oppose tumorigenesis in the setting of CAC. In a recent study highlighting the applicability of big data methods to such questions, investigators used a transcriptomic approach to find that*Mmp9* and *Mmp3* were overexpressed in colitis, but not in CAC (13). Using different approaches, others reported increased MMP-9 expression in CAC tissue samples (99, 117). Decades of investigation by Garg and colleagues uncovered potential mechanisms whereby MMP-9 regulates CAC by promoting caspase-3-dependent apoptosis, restraining cell proliferation, and limiting DNA damage by reactive oxygen species (ROS) (118, 119). MMP-9 most likely mediates enterocyte apoptosis and proliferation by activating Notch-1, a transcription factor which gatekeeps the differentiation of goblet cells and, in turn, activates the tumor suppressor p53 (120). In multiple models, inhibiting this pathway leads to increased tumor number and grade. Both *in vivo* and *in vitro*, MMP-9 was found to be involved in mismatch repair and amelioration of oxidative stress in CAC (119). Thus, although MMP-9 promotes inflammation in non-cancerous IBD via ECM degradation, by a different mechanism, it acts as a tumor suppressor in CAC. Furthermore, *Timp1*, which encodes a TIMP that preferentially inhibits MMP-1, -3, -7, and -9, is overexpressed in colitis and CAC, suggesting tight regulation of MMP activity in both disorders (13).

Other MMPs have been studied in the context of CAC, however their roles remain largely obscure. Compared to ulcerative colitis without dysplasia, MMP-7 expression is increased at the crypt bases of CAC tissue and its expression is more widespread in high-grade compared to low-grade dysplasia (93). It is possible that increased MMP-7 expression in CAC may result from the loss of *APC*, which occurs relatively later in the progression from inflammation-related dysplasia to CAC. APC loss results in nuclear translocation of β-catenin and activation of MMP-7 transcription via TCF-4, like the mechanism underlying MMP-7 overexpression in sporadic CRC (121). In a transcriptomics study, the genes encoding MMP-7 and MMP-13 were overexpressed in CAC but not in colitis. In contrast to pre-omics era MMP expression data, this difference might be attributable to the analysis of a different tissue type (13, 113). Additional studies using *in vivo* models of colitis and CAC found that the expression signature of colitis (i.e., *Mmp3* and *Mmp9*) versus that of CAC (i.e., *Mmp7* and *Mmp13*) could predict the development of CAC in mice with dextran sulfate sodium (DSS)-induced colitis (13).

Koller et al. found that MMP-10-deficient mice were more susceptible to DSS-induced colitis, a common experimental model approximating UC, and their disease was more severe and refractory to resolution. Moreover, MMP-10-deficient mice had a higher burden of inflammation-associated colonic dysplasia, suggesting that MMP-10 may protect against both colonic inflammation and CAC (103).

MMP-11 mRNA expression is also upregulated in CAC compared to normal tissue, and the positive correlation between MMP-11 and β-catenin expression in intestinal crypts suggests the mechanism may be similar to that for MMP-7, i.e., *APC* loss in CAC (117). Interestingly, in mice, treatment with the proton pump inhibitor omeprazole prevented experimental CAC, with concomitant decreases in MMP-11, MMP-9, and MMP-14 (aka MT1-MMP) and colon inflammatory markers. While attempting to induce experimental CAC, the same group found similar decreases in MMP-11 and -9 expression and activity in mice treated with infliximab, an anti-TNF-α medication used in IBD (105). Little else is known about the role of MMP-11 in CAC.

### 3.3 MMPs as potential biomarkers

The dynamic levels of selective MMP expression in inflammation and disease progression, including CRC, make them attractive biomarker candidates. Different methods of analysis such as ELISA, zymography, mass spectrometry, and cytology are used to detect MMPs in plasma, serum, urine, and tissue samples (122, 123). Incidentally, it is important to pay attention to sample collection – differences in MMP concentration values are reported depending on sample type (e.g., higher in serum compared to plasma) (124).

The utility of MMPs to mark disease status was studied in different contexts, including COVID-19 infection, cardiovascular disease, IBD, and cancer. In sera from 156 patients hospitalized with COVID-19 and respective controls, Gelzo et al. detected a significant increase in MMP-3 in early infection and increased MMP-9 levels throughout the course of disease (125). In a prospective study of 1127 patients with coronary artery disease, Blankenberg et al. reported an increase in plasma MMP-9 levels, deeming it a possible predictor of cardiovascular mortality (126). Regarding inflammation, Yoblecovitch et al. noted that serum MMP-9 levels predicted a clinical flare in patients with quiescent Crohn’s disease (127). Lastly, in CRC, elevated levels of MMP-2, MMP-9, and MMP-13 were observed in plasma and cancer biopsy samples (128–130). Together, these proofs of principle suggest that specific MMPs can be used to gauge disease status and intestinal inflammation, thus highlighting their potential use as clinical biomarkers.

## 4 Clinical therapies targeting MMPs in CAC

### 4.1 Approaches to targeting MMPs

MMPs are increasingly of interest as therapeutic targets for several disorders, including complications of diabetes (e.g., diabetic retinopathy (131) and foot ulcers (132)), and ischemic heart injury (133). Both natural (i.e., TIMPs) and synthetic MMP inhibitors have been pursued as therapeutic agents. In early studies, TIMPs were of great interest as natural inhibitors, particularly given their aberrant expression in multiple cancer types (134). Unfortunately, the lack of effective and selective delivery methods and increased TIMP levels in several cancers complicate their use. TIMP size and structure make tissue penetration and targeted delivery difficult, and once administered, they are prone to rapid degradation. Further, there is an inverse correlation between survival and TIMP levels. Although the rise in TIMP levels is likely due to a concurrent rise in MMP levels, their failure to impact tumor progression suggests that exogenous TIMPs are unlikely to have therapeutic efficacy (135), even though they may synergize with synthetic MMP inhibitors (MMPIs) (136). Neovastat, a compound derived from shark cartilage, targets MMP-2 while also blocking VEGF signaling. Neovastat has only been explored for non-GI metastatic cancers (e.g., breast, prostate, kidney, and lung (137)), but its dual mechanism of action increases the likelihood of success beyond phase III clinical trials. Combined with its excellent safety profile, these features suggest Neovastat may be an attractive candidate to target cancers in which MMP-2 is an important player (138).

Synthetic MMPIs are generally categorized as broad-spectrum peptidomimetics, non-peptidomimetics, tetracycline derivatives, and bisphosphonates (139). Peptidomimetics are collagen-mimicking pseudopeptides that gained traction as potential inhibitors, though they have not yet succeeded in phase III clinical trials. The archetype, Marimastat, efficaciously targets MMP-1, -3, -7, and -9 and dose-dependently reduced carcinoembryonic antigen (CEA) levels in recipients with CRC (140). Although Marimastat’s excellent oral bioavailability and success in phase III trials were favorable, the lack of extended overall survival and continued tumor progression diminished enthusiasm (139). Synthetic MMPIs may provide benefit when used in combination with other therapies; for example, TIMP-2 synergistically enhances the effects of Marimastat (136).

Synthetic non-peptidomimetics, described in more detail below, provide an alternative therapeutic avenue. Their development resulted from an attempt to increase oral bioavailability and improve pharmacokinetic properties of peptidomimetic compounds (141). Many are specific to MMP-2, -3, and -9, such as Prinomostat’s targeting of MMP-2, -3, -9, -13, and -14 and Tanomastat’s targeting of MMP-2, -3, and -9 (142). Tetracycline derivatives, such as Periostat and Metastat, inhibit both the production and secretion of MMP-2 and -9 (143, 144), however, because of their toxicity at therapeutic doses, greater tissue specificity is required for successful drug development.

Another approach utilizes miRNAs to modify the action of MMPs indirectly. Multiple potential points of intervention exist, including at the level of MMP secretion, enzymatic activity, and expression. An example pursued in sporadic CRC is miR-34a, a miRNA downregulated in several digestive cancers that transcriptionally targets p53 (60). Treatment of colon cancer cells with miR-34a reduced MMP-1 and -9 protein levels and decreased proliferation and invasion *in vitro* (60). However, targeted delivery of miRNAs remains challenging. A potential strategy is to target tissues that exhibit increased MMP activity using polyplexes of miRNA and MMP-responsive polyethylene glycol (PEG) shields, which can deshield in the presence of high MMP levels. miR-34a has been successfully polyplexed to MMP-2-responsive PEGylated polyethyleneimine (PEI), a complex in which PEG shields PEI, but can be cleaved by MMP-2. Compared to treatment with non-PEGylated PEI and miRNA, application of this MMP-cleavable system increases cellular co-uptake of the PEGylated PEI and miRNA, and improves metrics of anti-cancer activity *in vitro* and *in vivo* (145, 146).

### 4.2 Synthetic MMP inhibitors

The 1990s witnessed great interest in the therapeutic potential of synthetic MMPIs for cancer. Theoretically, synthetic MMPIs offered a narrower spectrum of MMP inhibition compared to their predecessors, and might therefore target specific MMPs and avoid off-target toxicity. The earlier synthetic MMPIs were typically small molecule inhibitors which targeted the MMP active site using zinc binding groups on a peptide backbone (147). Aside from poor MMP selectivity, low efficacy and musculoskeletal side effects prevented these small molecule inhibitors, including Batimastat and Marimastat, from advancing through clinical trials for CRC (148, 149). Later, the use of transition state analogs and of targeting multiple MMP structural features, e.g., the variable S1’, S2, and S3 subsites and exosite domains, improved substrate selectivity (148, 150, 151).

In the early 2010s, another avenue in MMPI drug development emerged: allosteric regulation. In a proof of principle, Udi et al. developed two branched amphiphilic molecules capable of binding to “hidden regulatory sites” unique to MMP-12 and MMP-14, thereby disrupting the geometric conformation of these MMPs and their function, without the need for a less specific zinc binding group (152). Soon thereafter, synthetic allosteric inhibitors and inhibitory antibodies were developed to target MMPs implicated in disease. In a murine model, AB0041, an antibody which noncompetitively inhibits MMP-9 with high specificity, attenuated colon cancer xenograft growth and metastasis (153). The humanized form of AB0041, GS-5745 (Andecaliximab), had equivalent potency and selectivity and completed Phase I clinical trials for UC and advanced solid tumors, including CRC, but has not been studied in CAC (153–155). Notably, Andecaliximab is the only MMP-targeting monoclonal antibody that has undergone clinical trials (149). As noted by others, MMPs may be more active in the pre- and peri-metastatic phases of disease. Hence, studying MMPIs only in advanced (i.e., metastatic) cancer may miss interventional opportunities; perhaps including early-stage cancers in MMPI trials would yield more favorable results (156).

### 4.3 Challenges in clinical application

Promising avenues for the use of MMP inhibitors in CRC treatment are well established, but general challenges in their clinical use warrant discussion. As discussed in section 2, the development of specific MMP inhibitors was complicated by the hard-to-define role certain MMPs had in inflammatory disease progression, along with general substrate redundancy and high structural homology (147). Broad spectrum MMPI use encountered challenges such as low bioavailability, a poor metabolic profile, and, as in the case of Marimastat, limiting musculoskeletal side effects (157). Administration of non-pepdomimetic compounds also led to variable effects in cell proliferation and adverse reactions. For example, in phase III clinical trials for non-small lung cancer, determining the dosage and timing of Tanomastat administration proved problematic (158). Tetracycline derivatives such as Metastat, trialed for advanced solid tumors, were associated with severe toxicity, including photosensitivity skin reactions, showing that greater tissue selectivity is required (159).

Over time, more selective MMPIs were developed via NMR and X-ray crystallography methods to identify less conserved binding sites, such as cavities formed specific to individual enzymatic structures (158). As novel MMPIs progress through clinical trials, fewer challenges pertaining to delivery or heterogeneous effects are noted. For example, in a randomized phase I/II study in psoriasis, the adverse effects of Neovastast, used to inhibit MMP-2 and VEGF, were mainly nausea and diarrhea (160). As observed in phase Ib and phase II/III clinical studies for ulcerative colitis and rheumatoid arthritis, selective MMP-9 inhibition using the monoclonal antibody Andecaliximab also has a generally safer side-effect profile. In these trials, test subjects exhibited modest adverse events requiring only observation or minimal interventions (161, 162). As we consider challenges in their therapeutic use, in addition to efficacy and safety concerns, the high cost of monoclonal antibodies and similar advanced approaches should be considered.

## 5 Conclusions and future directions

As a consequence of chronic inflammation, colitis increases the risk of developing CAC, underscoring the importance of understanding the pathology of chronic inflammation-induced CRC (163). Although previously thought to participate only in ECM degradation, MMPs are now recognized to be directly involved in the development and sustenance of inflammatory states, including those that underlie both Crohn’s and ulcerative colitis. Yet, despite advances reviewed here, large gaps in knowledge exist regarding the role MMPs play in both CAC and the chronic inflammation that precedes and promotes CAC. There is a concordant lack of information regarding the potential utility of MMP inhibitors to treat these disorders.

The upregulation of specific MMPs, like MMP-1, -2, -3, and -7, in intestinal inflammation and IBD implicates not only their contribution to the development of CAC but also their potential as biomarkers to identify increasing dysplasia and incipient neoplasia. In contrast, other MMPs, like MMP-10, appear to be protective, auguring a more favorable prognosis. Some, like MMP-9, play seemingly context-dependent dual pro-inflammatory and anti-tumorigenic roles. Thus, prospectively testing the value of individual MMPs or a panel of MMPs in blood and tissue samples to gauge their value as biomarkers for dysplasia and incipient neoplasia appears to be a worthy avenue of investigation.

This comprehensive review of MMP involvement in CAC pathogenesis and potential therapeutic approaches targeting MMPs, emphasizes the need for continuing research focused on unraveling the pathways whereby different MMPs exert their actions. For example, further work is needed to unravel the pathways that modulate selective MMP upregulation after muscarinic agonism in colon cancer, how this interplay contributes to an invasive phenotype, and its importance for the development and progression of CAC. Another potential avenue is to use this information to develop a panel of MMPs that can be tested in primary tumors to gauge the potential for advancing disease, the need for adjuvant therapy, and the likelihood of response to conventional chemotherapeutic regimens. Lastly, continuing attention must be devoted to exploring the growing therapeutic applications of MMP inhibitors, particularly as they relate to the management of IBD and CAC in those with an MMP profile predictive of aggressive disease.

## Author contributions

NS: Conceptualization, Investigation, Writing – original draft, Writing – review & editing. AS: Conceptualization, Investigation, Writing – original draft, Writing – review & editing. MA: Data curation, Writing – original draft, Writing – review & editing. SP: Data curation, Writing – original draft. J-PR: Conceptualization, Data curation, Funding acquisition, Writing – original draft, Writing – review & editing.
